# 2343. SARS-CoV-2 Spike Conformation Essentially Influences Vaccine Immunogenicity – Results from Two Clinical Phase I Trials of the Vector Vaccine Candidates MVA-SARS-2-S and MVA-SARS-2-ST

**DOI:** 10.1093/ofid/ofad500.1965

**Published:** 2023-11-27

**Authors:** Anahita Fathi, Christine Dahlke, Svenja Hardtke, Sibylle C Mellinghoff, Leonie Mayer, Leonie M Weskamm, Verena Krähling, Helena Müller-Kräuter, My L Ly, Monika Friedrich, Thomas J Hesterkamp, Asisa Volz, Gerd Sutter, Stephan Becker, Marylyn Addo

**Affiliations:** University Medical Center Hamburg-Eppendorf, Hamburg, Hamburg, Germany; University Medical Center Hamburg-Eppendorf, Hamburg, Hamburg, Germany; University Medical Center Hamburg-Eppendorf, Hamburg, Hamburg, Germany; University Hospital Cologne, Cologne, Nordrhein-Westfalen, Germany; University Medical Center Hamburg-Eppendorf, Hamburg, Hamburg, Germany; University Medical Center Hamburg-Eppendorf, Hamburg, Hamburg, Germany; Philipps University Marburg, Marburg, Hessen, Germany; Philipps University Marburg, Marburg, Hessen, Germany; University Medical Center Hamburg-Eppendorf, Hamburg, Hamburg, Germany; University Medical Center Hamburg-Eppendorf, Hamburg, Hamburg, Germany; German Center for Infection Research, Braunschschweig, Niedersachsen, Germany; University of Veterinary Medicine Hannover, Hanover, Niedersachsen, Germany; Ludwig Maximillian University Munich, Munich, Bayern, Germany; Philipps University Marburg, Marburg, Hessen, Germany; I. Department of Medicine, University Medical Center Hamburg-Eppendorf, Hamburg, Germany, Hamburg, Hamburg, Germany

## Abstract

**Background:**

Recombinant Modified Vaccinia virus Ankara (MVA) is a replication-deficient viral vector platform with a favorable safety profile that is currently developed for multiple indications, including vaccines against emerging infections. We assessed the safety and immunogenicity of two MVA-based vaccine candidates against COVID-19 encoding the native or pre-fusion stabilized SARS-CoV-2 spike antigen, namely MVA-SARS-2-S and MVA-SARS-2-ST, in two clinical Phase I trials.

**Methods:**

We conducted a first-in-human trial of the vaccine candidate MVA-SARS-2-S (NCT04569383) and a Phase Ib trial of the successor vaccine MVA-SARS-2-ST (NCT05226390) in healthy, SARS-CoV-2 immune-naïve adults aged between 18-55 (MVA-SARS-2-S) and 18-64 (MVA-SARS-2-ST) years. MVA-SARS-2-S expresses the native, full-length spike glycoprotein of SARS-CoV-2 (S), while MVA-SARS-2-ST delivers a pre-fusion stabilized spike antigen (ST). Both trials were designed as open-label dose-escalation trials and vaccinations were administered as a prime-boost regimen on days 0 and 28. Detailed safety and immunogenicity assessments were conducted at multiple timepoints throughout the study until day 168.

**Results:**

In NCT04569383, 30 individuals received either 1 x 10^7^ ± 0.5 log IU (IU=International Units, n=15, lower dose) or 1 x 10^8^ ± 0.5 log IU (n=15, higher dose) of MVA-SARS-2-S. In NCT05226390, 8 participants received a lower (≥ 1 x 10^7^ IU) and 7 participants a higher (≥ 5 x 10^7^ IU) dose of MVA-SARS-2-ST. Both vaccines were well-tolerated and no vaccine-related severe adverse events (SAEs) occurred in either trial. However, seroconversion rates, measured as binding anti-SARS-CoV-2 S1 antibodies, could only be observed in 3/15 participants in the high dose cohort and none in the low dose cohort after vaccination with MVA-SARS-2-S. In contrast, all participants who received MVA-SARS-2-ST (n=15) achieved seroconversion independent of administered dose.

**Conclusion:**

Both vaccine candidates were safe and well-tolerated. While MVA-SARS-2-S did not induce adequate immunogenicity, vaccination with the pre-fusion stabilized successor vaccine MVA-SARS-2-ST induced seroconversion in all vaccinees, demonstrating the significance of SARS-CoV-2 spike antigen conformation for vaccine immunogenicity.

**Disclosures:**

**Anahita Fathi, MD**, Biotest: Honoraria **Sibylle C. Mellinghoff, MD**, Gilead: Grant|Octapharma: Advisor/Consultant

